# Carbon monoxide-induced TFEB nuclear translocation enhances mitophagy/mitochondrial biogenesis in hepatocytes and ameliorates inflammatory liver injury

**DOI:** 10.1038/s41419-018-1112-x

**Published:** 2018-10-17

**Authors:** Hyo Jeong Kim, Yeonsoo Joe, So-Young Rah, Seul-Ki Kim, Se-Ung Park, Jeongmin Park, Jin Kim, Jinhyun Ryu, Gyeong Jae Cho, Young-Joon Surh, Stefan W. Ryter, Uh-Hyun Kim, Hun Taeg Chung

**Affiliations:** 1 Department of Biological Sciences, University of Ulsan, Ulsan, 680-749 Republic of Korea; 2 National Creative Research Laboratory for Ca2+ Signaling Network, Chonbuk National University Medical School, Jeonju, Republic of Korea; 3 Department of Anatomy, School of Medicine and Institute of Health Sciences, Gyeongsang National University, JinJu, 660-701 Republic of Korea; 4 Tumor Microenvironment Global Core Research Center and Research Institute of Pharmaceutical Sciences, College of Pharmacy, Seoul National University, Seoul, Republic of Korea; 5 Division of Pulmonary and Critical Care Medicine, Joan and Sanford I. Weill Department of Medicine, Weill Cornell Medical Center, New York, NY USA

## Abstract

Carbon monoxide (CO) can confer protection against cellular stress, whereas the potential involvement of autophagy and lysosomal biogenesis remains incompletely understood. We demonstrate here that the activation of protein kinase R (PKR)-like endoplasmic reticulum (ER) kinase (PERK) with CO increased the nuclear translocation of transcription factor EB (TFEB). PERK activation by CO increased intracellular Ca^2+^ concentration and the phosphatase activity of calcineurin against TFEB. Moreover, we found that in the deficiency of TFEB, CO not only failed to recruit Parkin to the mitochondria but also failed to increase expression of lysosomal genes such as Lamp1, CathB, and TPP1. Therefore, we suggest that CO increases mitophagy through TFEB nuclear translocation by PERK-calcinuerin activation. In addition, the inhibition of TFEB with siRNA against TFEB abrogated the increase of mtDNA with CO, markers of mitochondrial biogenesis such as PGC1α, NRF1, and TFAM, and the mitochondrial proteins COX II, COX IV, and cytochrome c. To investigate the effects of CO on mitochondrial homeostasis in vivo, mice were treated with lipopolysaccharide (LPS)/d-galactosamine (D-GalN). CO inhalation reduced liver injury after challenge with LPS/GalN. Furthermore, CO inhalation increased TFEB activation, mitophagy and mitochondrial biogenesis in mice treated with LPS/GalN. Our findings describe novel mechanisms underlying CO-dependent cytoprotection in hepatocytes and liver tissue via activation of TFEB-dependent mitophagy and associated induction of both lysosomal and mitochondrial biogenesis.

## Introduction

Autophagy is a genetically regulated cellular homeostatic program for the lysosome-dependent clearance of misfolded proteins, defective mitochondria and other organelles, lipid droplets, damaged DNA, and invading pathogens^1,2^. Transcription factor EB (TFEB) is a master regulator of the autophagy/lysosomal pathway, which is activated in response to multiple stimuli including endoplasmic reticulum (ER) stress, mitochondrial stress, and pathogen exposure, and which can regulate protein folding, mitochondrial homeostasis, and immune responses^3–5^. During starvation, inactivation of mechanistic target of rapamycin (mTOR) complex 1 (mTORC1) in combination with activation of the Ca^2+^-dependent phosphatase calcineurin promotes TFEB dephosphorylation and nuclear translocation. The activated TFEB stimulates autophagy and induces the expression of lysosomal genes by binding to the coordinated lysosomal expression and regulation (CLEAR) element in the regulatory regions of its target genes, and also increases the expression of master regulators of lipid catabolism such as peroxisome proliferator-activated receptor-γ coactivator 1-α (PGC1α) and peroxisome proliferator-activated receptor-α^6–8^.

The activation or overexpression of TFEB has been shown to promote the clearance of mutant SERPIN1A protein in hepatic disease, suppressing disease phenotype^9^. Furthermore, liver-specific genetic deletion of TFEB results in the accumulation of lipid droplets and defective lipid degradation during starvation. Conversely, overexpression of TFEB enhances fatty acid catabolism, while inhibiting obesity and metabolic syndrome in high-fat diet-fed mice^10^. Therefore, manipulation of TFEB-mediated autophagy and lysosomal biogenesis may provide therapeutic benefit in metabolic diseases. Currently, pharmacological agents that modulate TFEB activity remain scarce.

To maintain proper mitochondrial homeostasis, the removal of damaged mitochondria by mitophagy must be tightly regulated and counterbalanced by mitochondrial biogenesis^11^. Accumulation of dysfunctional mitochondria as the result of insufficient mitophagy may constitute a signal for inflammasome activation^12^. Downregulation of TFEB causes impaired autophagic flux, degradation of damaged mitochondria, and increases in myocardial oxidative stress^13^. TFEB promotes mitochondrial degradation and biogenesis by upregulating PGC1α expression, a master regulator of mitochondrial biogenesis^4^. Hence, TFEB may establish a positive feedback loop that maintains the balance between mitophagy and mitochondrial biogenesis.

Carbon monoxide (CO) is an endogenously produced gaseous molecule that is generated endogenously via the catabolism of heme by heme oxygenase (HO) enzymes, which include the inducible isozyme HO-1. The HO-1/CO system responds to induction by oxidative stress, hypoxia, hyperoxia, hypothermia, ER stress, inflammation, and ischemia^14,15^. Low concentrations of CO can protect hepatocytes against apoptosis and also exerts an anti-inflammatory function via increased generation of mitochondrial (mt) reactive oxygen species (ROS), which are crucial for signaling^16,17^. Recent studies have shown that a low concentration of mtROS generated by mild mitochondrial dysfunction and calorie restriction results in increased lifespan of *Caenorhabditis elegans* and of mice by triggering defense mechanisms that prevent cellular damage^18^. Although high concentrations of ROS can cause severe oxidative damage, a moderate increase in ROS may serve as a signal to trigger autophagy and other cell survival mechanisms^19^. The stimulation of low levels of mtROS as signaling molecules by CO likely results in augmented stress defense mechanisms. In the present study, we investigated whether CO, which activates PERK signaling via mtROS generation, can activate calcineurin-dependent TFEB nuclear translocation, and subsequent mitophagy and mitochondrial biogenesis. Our data indicate that CO ameliorates acute hepatitis-induced liver injury by preserving mitochondrial homeostasis. We conclude that CO can stimulate TFEB activation, which coordinates mitophagy and mitochondrial biogenesis to ensure mitochondrial quality and prevent tissue injury associated with mitochondrial dysfunction. Our results lend further support to the potential therapeutic application of CO in hepatic diseases.

## Results

### CO increases TFEB nuclear translocation via the PERK-Ca^2+^-calcineurin pathway

The PERK-dependent activation of TFEB contributes to cellular adaptation to stress by inducing autophagy genes^3^. We have previously reported that the HO-1/CO system protects cells against ER stress via activating the PERK-dependent pathway and conveying ER stress to cell survival signals from the mitochondria^20,21^. To confirm the relationship between CO-activated PERK and the activation of TFEB, we first examined whether CO-releasing molecules CORM2 can induce TFEB nuclear translocation.

**Fig. 1:**
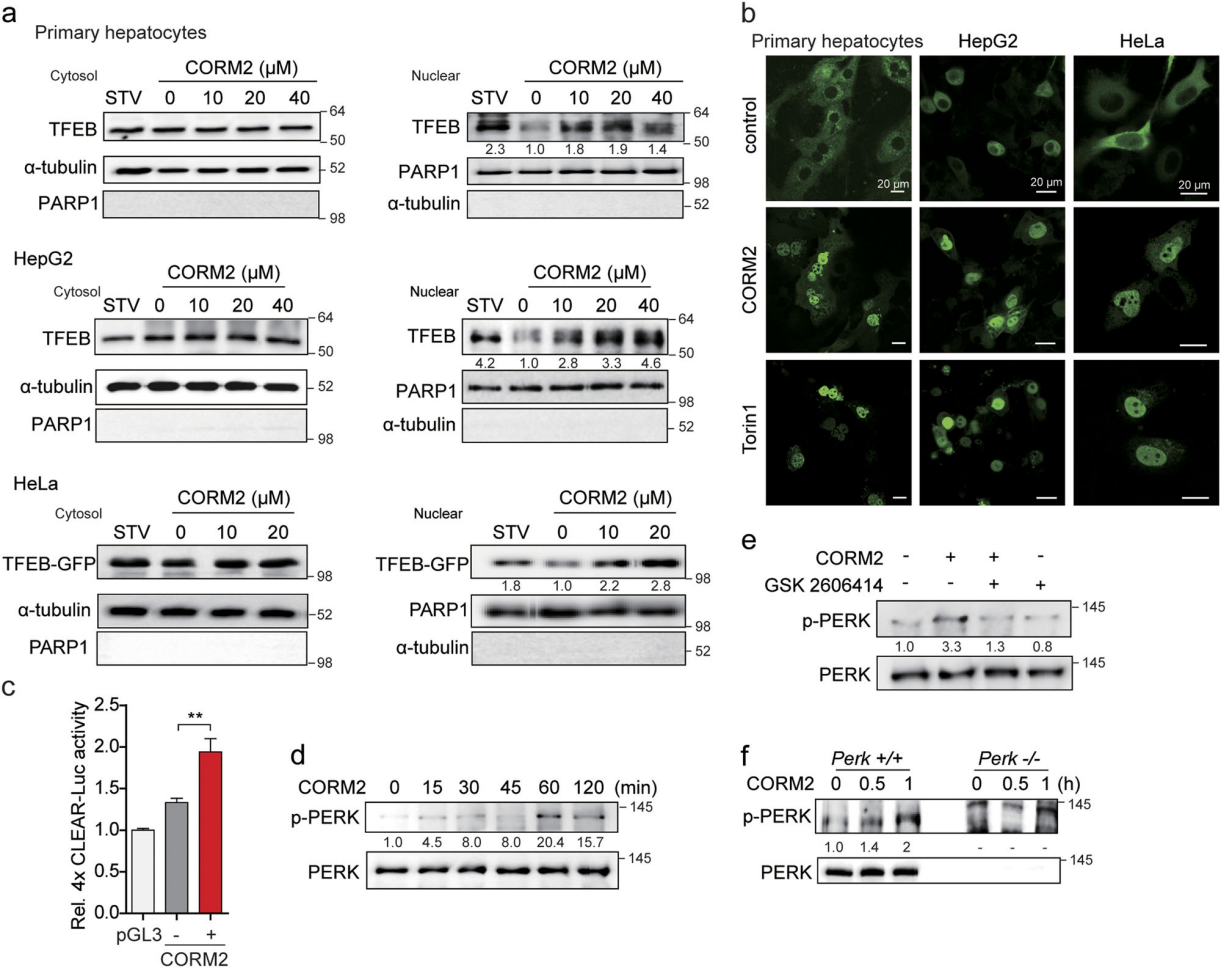
Carbon monoxide (CO) increases TFEB nuclear translocation. **a** Primary hepatocytes, HepG2, or HeLa cells were incubated with CORM2 at the indicated doses or starved (STV) for 3 h. Cells were fractionated into cytosol and nuclear. Endogenous TFEB proteins were analyzed by immunoblotting. HeLa cells were transfected with TFEB-GFP plasmid for 24 h and then treated with CORM2 at the indicated dose for 3 h. Nuclear translocation of CORM2-induced TFEB-GFP was detected by anti-GFP antibody. **b** Primary hepatocytes, HepG2, and HeLa cells were transfected with TFEB-GFP plasmid for 24 h. After transfection, cells were treated with CORM2 (20 μM) or Torin 1 (1 μM) for 3 h and TFEB localization was determined by confocal fluorescence microscopy. Scale bar, 20 μm. **c** Primary hepatocytes were co-transfected with 4X CLEAR-luciferase reporter construct and pRL-SV40 *Renilla* luciferase construct for 24 h. After treatment with CORM2 for 9 h, cells were lysed and assayed for luciferase activity. Values are shown as mean ± SEM (*n* = 3). ***P < 0.001. **d** Primary hepatocytes were treated with CORM2 (20 μM) for the indicated times. The levels of phosphorylated PERK (p-PERK) and total PERK were measured by immunoblotting. **e** Primary hepatocytes were pretreated with GSK2606414 (PERK inhibitor, 0.5 μM) for 30 min and then treated with CORM2 (20 μM) for 1 h. The levels of phosphorylated PERK (p-PERK) and total PERK were measured by immunoblotting. **f** *Perk*^*+/+*^ and *Perk*^−/−^ MEF cells were treated with CORM2 (20 μM) for 30 min and 1 h. The levels of phosphorylated PERK (p-PERK) and total PERK were measured by immunoblotting. Band intensities were determined by densitometry (Image J). Basal levels in the untreated sample were set at 1.0 and results are expressed as fold induction over control levels

We found that CORM2 stimulated the increased expression of endogenous TFEB in nuclear fractions of primary hepatocytes or HepG2, and also increased the expression of exogenous GFP-tagged TFEB in the nuclear fractions of transfected HeLa cells (Fig. 1a). Consistently, confocal analysis revealed increased nuclear translocation of GFP-tagged TFEB in primary hepatocytes, HepG2, and HeLa cells in response to CORM2 treatment (Fig. 1b). The mTORC1 inhibitor Torin 1 also elicited similar nuclear translocation of TFEB in these cells. Furthermore, CORM2 treatment increased luciferase activity of a transfected construct containing a tetrameric repeat of the CLEAR motif, a target of TFEB binding (Fig. 1c and Supplementary Figure S1a).

We next investigated the mechanisms underlying the CO-dependent regulation of TFEB nuclear translocation and the involvement of Ca^2+^ mobilization mediated by the ER stress kinase PERK. [Ca^2+^]i elevation in myocytes is regulated by PERK/calcineurin-mediated dissociation of the FK506-binding protein (FKBP12.6) from the ryanodine receptor^22^. During cellular stress, TFEB activity is regulated by the calmodulin-regulated protein phosphatase calcineurin, which mediates the dephosphorylation of TFEB at specific residues^3,23^. We first confirmed that CORM2 increased PERK phosphorylation in a time-dependent manner (Fig. 1d, Supplementary Figure S1b), whereas PERK inhibitor GSK2606414 suppressed CO-induced PERK phosphorylation (Fig. 1e, Supplementary Figure S1c). As expected, CO-dependent PERK phosphorylation was absent in PERK knockout (*Perk*^−/−^) MEF relative to PERK wild-type (*Perk*^*+/+*^) MEF (Fig. 1f). These results confirm that CO can activate PERK signaling. In addition, we investigated whether PERK activation by CO was associated with mitochondrial ROS using the mitochondria-targeted antioxidant mito-TEMPO. CORM2 increased mitochondrial ROS production, as detected with MitoSOX Red (Supplementary Figure S1d). CORM2 activates PERK phosphorylation and this effect was inhibited by Mito-TEMPO (Supplementary Figure S1e). These results confirm that CO can activate PERK signaling through generation of low levels of mitochondrial ROS.

**Fig. 2:**
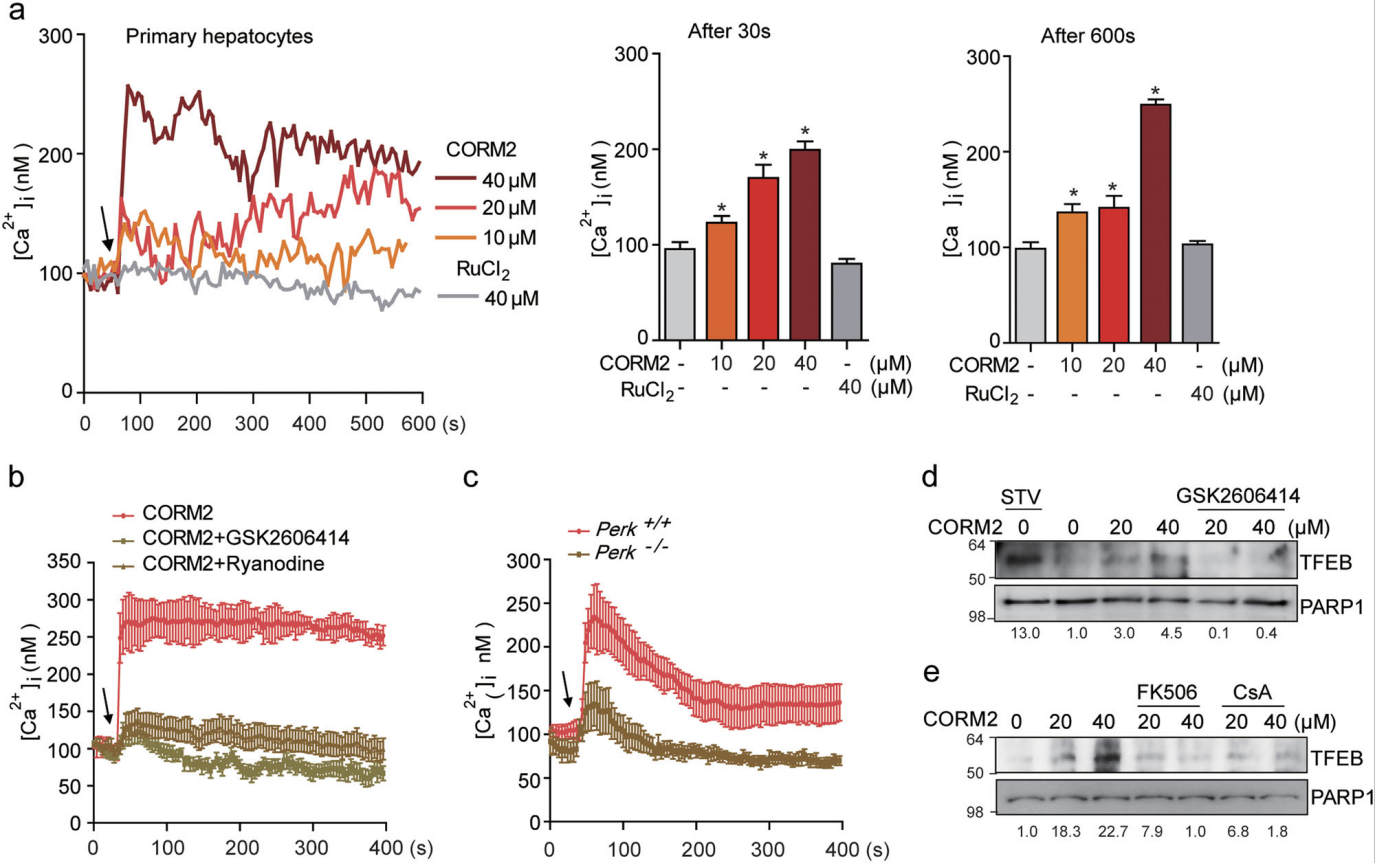
CO increases TFEB nuclear translocation through PERK-Ca^2+^-calcineurin pathway. **a** Left panel, primary hepatocytes were pretreated with fluo-4 AM and the change of [Ca^2+^]_i_ was measured using confocal microscopy. The arrow indicates the time point at which CORM2 or ruthenium chloride (control) was added. The data show the mean ± SD from three independent experiments. Middle panel, comparison of mean [Ca^2+^]_i_ 30 s after treatment with CORM2 or ruthenium chloride. Data are mean ± SD from three independent experiments. *P < 0.001 vs. basal. Right panel, comparison of mean [Ca^2+^]_i_ 600 s after the treatment with CORM2 or ruthenium chloride. The data are mean ± SD from three independent experiments. *P < 0.001 vs. basal. **b** GSK2606414 (PERK inhibitor, 1 μM) or Ryanodine (20 μM) was preincubated for 30 min. Arrow indicates the time point at which 30 μM CORM2 was added. Data are mean ± SD from three independent experiments. **c** CORM2-induced Ca^2+^ increase was blocked in PERK KO MEF cells. The data show the mean ± SD from three independent experiments. **d** Primary hepatocytes were pretreated with GSK2606414 for 30 min and then cells were treated with CORM2 or tunicamycin (TM). Endogenous nuclear TFEB was analyzed by immunoblotting. **e** Primary hepatocytes were incubated with CORM2 or starved for 3 h in the presence or absence of calcineurin inhibitor, FK506 (5 μM), or cyclosporin A (5 μM) for 1 h. Band intensities were determined by densitometry (Image J). Basal levels in the untreated sample were set at 1.0 and results were expressed as fold induction over control levels

As the HO-1/CO system is known to stimulate l-type calcium channel-mediated Ca^2+^ influx in astrocytes^24^, we examined the effects of CO on cytosolic Ca^2+^ levels. As shown in Fig. 2a, CORM2 treatment elicited a rapid dose-dependent increase in Ca^2+^ levels in hepatocytes, relative to ruthenium chloride, a control for the CORM backbone. We next assessed whether PERK mediates the regulation of Ca^2+^ levels in response to CO stimulation. Pretreatment with GSK2606414, a PERK inhibitor, abrogated CO-induced Ca^2+^ signals (Fig. 2b). Also, inhibition of RyR channel activity with ryanodine significantly suppressed CO-induced Ca^2+^ levels (Fig. 2b). Therefore, we postulated that CO-induced Ca^2+^ levels might be triggered by calcineurin-mediated disassociation of the FKBP from the RyR. *Perk*^−/−^ MEF also displayed significantly lower Ca^2+^ levels compared with *Perk*^*+/+*^ MEF when treated with CORM2 (Fig. 2c). In addition, we analyzed whether CO-generated mtROS affects TFEB nuclear translocation. CO-induced TFEB nuclear translocation was inhibited by mito-TEMPO, indicating CO affects the TFEB (Supplementary Figure S1f).

The inhibition of PERK with GSK2606414 significantly decreased CO-induced TFEB nuclear translocation (Fig. 2d and Supplementary Figures S1g). Given that intracellular Ca^2+^ elevation promotes TFEB nuclear translocation through the activation of calcineurin^23^, we additionally investigated whether CO-induced Ca^2+^ levels promotes TFEB nuclear translocation through calcineurin activity. Treatment with FK506 or cyclosporine A (CsA), an inhibitor of calcineurin activity, remarkably abolished CO-induced TFEB nuclear translocation (Fig. 2e and Supplementary Figure S1h). Our data suggest that CO promotes TFEB nuclear translocation through PERK-dependent Ca^2+^ signaling and calcineurin activation.

### CO enhances mitophagy via TFEB activation with lysosomal biogenesis

**Fig. 3:**
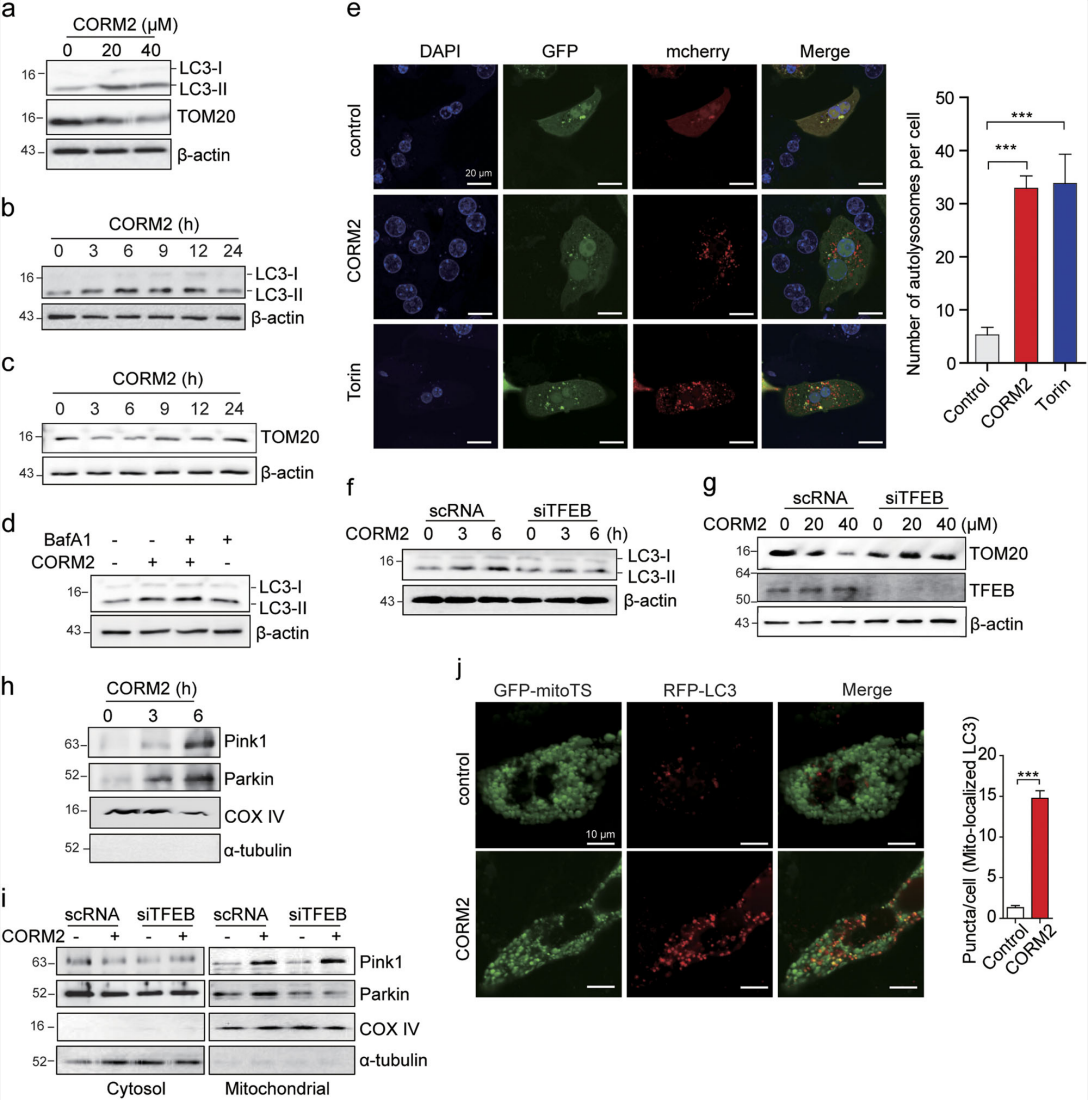
CO enhances mitophagy through TFEB activation. **a–c** Primary hepatocytes were treated with CORM2 for 6 h at the indicated concentrations (**a**) or for the indicated times at 20 μM (**b** and **c**). The levels of LC3B-II and TOM20 were determined by immunoblotting. **d** Primary hepatocytes were treated with CORM2 (20 μM) in the presence of bafilomycin A1 (Baf A1), autophagy flux inhibitor. The cells were collected and cell lysates were subjected to immunoblotting to detect the expression of LC3B. **e** Primary hepatocytes were transfected with mcherry-GFP-LC3 for 24 h. After transfection, cells were treated with CORM2 (20 μM) or Torin 1 (1 μM) for 6 h, and analyzed by confocal fluorescence microscopy. Scale bar, 20 μm. The number of autolysosomes (red puncta) per cell (*n* = 10) was counted. Data are shown as mean ± SEM. ***P < 0.001 vs. control. **f**, **g** Primary hepatocytes were transfected with scrambled RNA (scRNA) or siRNA against TFEB (siTFEB) for 24 h, and then treated with CORM2 (20 μM) for the indicated times (**f**) or at the indicated doses for 6 h (**g**). The levels of LC3B-II, TOM20, and TFEB were determined by immunoblotting. **h** Mitochondrial fractions isolated from primary hepatocytes treated with CORM2 (20 μM) for the indicated times were analyzed by immunoblotting using antibodies against PINK1 and Parkin. **i** HepG2 were transfected with scRNA or siTFEB for 24 h and then treated with CORM2 (20 μM) for 6 h. Cells were fractionated into cytosol and mitochondria, and the levels of PINK1 and Parkin were analyzed by immunoblotting. Fraction quality was verified by immunoblotting with markers for the cytoplasm (α-tubulin) and mitochondria (cytochrome IV oxidase, COX IV). **j** Left panel, primary hepatocytes were transfected with GFP-mitoTS and RFP-LC3 for 24 h. After transfection, cells were treated with CORM2 (20 μM) for 3 h and analyzed by confocal fluorescence microscopy. Scale bar, 10 μm. Right graph, GFP-LC3 punctate structures associated with mitochondria were quantified. Values represent mean number of puncta counts per cell, *n* = 20 cells from two independent experiments. ***P < 0.001 vs. control

CO has been shown to induce autophagy through augmented mtROS production^25,26^. The increased production of mtROS at low levels may regulate mitochondrial quality by balancing mitochondrial autophagy (mitophagy) with mitochondrial biogenesis. First, to examine whether CO can activate autophagy in hepatocytes, we exposed hepatocytes to CORM2. Treatment with CORM2 increased the expression of the autophagy marker LC3B-II in a dose- or time-dependent manner (Fig. 3a, b and Supplementary Figure S2a), whereas the expression of mitochondrial protein translocase of outer membrane-20 (TOM20) was decreased up to 6 h and then gradually increased up to 24 h (Fig. 3c, Supplementary Figures S2a, b). To determine whether CO-induced accumulation of LC3B-II was caused by enhanced formation of autophagic vacuoles, or the blockage of autophagic vacuole degradation, hepatocytes were treated with CORM2 in the absence or presence of bafilomycin A1, an inhibitor of lysosomal degradation. The CO-dependent elevation in LC3B-II levels was further increased after treatment with bafilomycin A1, indicative of active autophagic flux in these cells (Fig. 3d and Supplementary Figure S2c). To further clarify whether CO increases autolysosome and autophagic flux, we used a tandem fluorescent-tagged LC3 expression plasmid, mCherry-GFP-LC3, expressing two fluorophores that display different pH sensitivities. As shown in Fig. 3e and Supplementary Figure S2d, cells treated with CORM2 displayed an increase in both GFP-LC3 and mCherry-LC3 fluorescent puncta, similar to cells treated with Torin 1, an mTOR inhibitor that increases autophagy. In the merged image, yellow puncta from colocalization of GFP-LC3 and mCherry-LC3 indicate autophagosome localization. CORM2 or Torin 1 increased the number of GFP-LC3^−^/mCherry-LC3^+^ puncta (red), as GFP is degraded in the lysosome acidic environment. This suggests that CORM2 increased autophagosme–lysosome fusion. These results suggest that autophagic flux was significantly increased in response to CORM2, similar to the response observed with Torin 1 used as a positive control.

To examine whether CO-activated autophagy flux requires TFEB, we used siRNA to knock down TFEB expression (Supplementary Figures S2e). As expected, TFEB-targeted siRNA (siTFEB) significantly abolished CO-induced LC3B-II levels (Fig. 3f and Supplementary Figures S2f). In contrast, CO-induced autophagic degradation of mitochondrial protein (TOM20) was inhibited by siTFEB, suggesting that CO promotes autophagy flux through TFEB (Fig. 3g and Supplementary Figure S2g). CO significantly increased the levels of mitophagy regulator proteins PINK1 and Parkin to the mitochondrial fraction of cells (Fig. 3h and Supplementary Figure S2h). In addition, we investigated whether CO-induced the cellular enrichment of mitochondrial PINK1 and Parkin is dependent on TFEB, using siRNA to knock down TFEB. After CO treatment, similar PINK1 levels were observed in cells transfected with either control siRNA (scRNA) or siTFEB. However, the CORM2-induced mitochondrial recruitment of Parkin was significantly decreased in TFEB knockdown cells relative to that of scRNA transfected cells (Fig. 3i). These results indicate that although CO can stimulate the enrichment of both mitochondrial PINK1 and Parkin, the mitochondrial recruitment of Parkin by CO was dependent on TFEB, whereas that of PINK1 occurred independently of TFEB. Moreover, mitophagosome formation by merge of RFP-LC3 puncta with GFP-mitoTS, containing mitochondrial targeting sequence as a mitochondrial marker, was significantly increased in CORM2-treated cells (Fig. 3j). Thus, these data revealed that CO induces Parkin recruitment to the mitochondria through TFEB, promoting mitophagy.

**Fig. 4:**
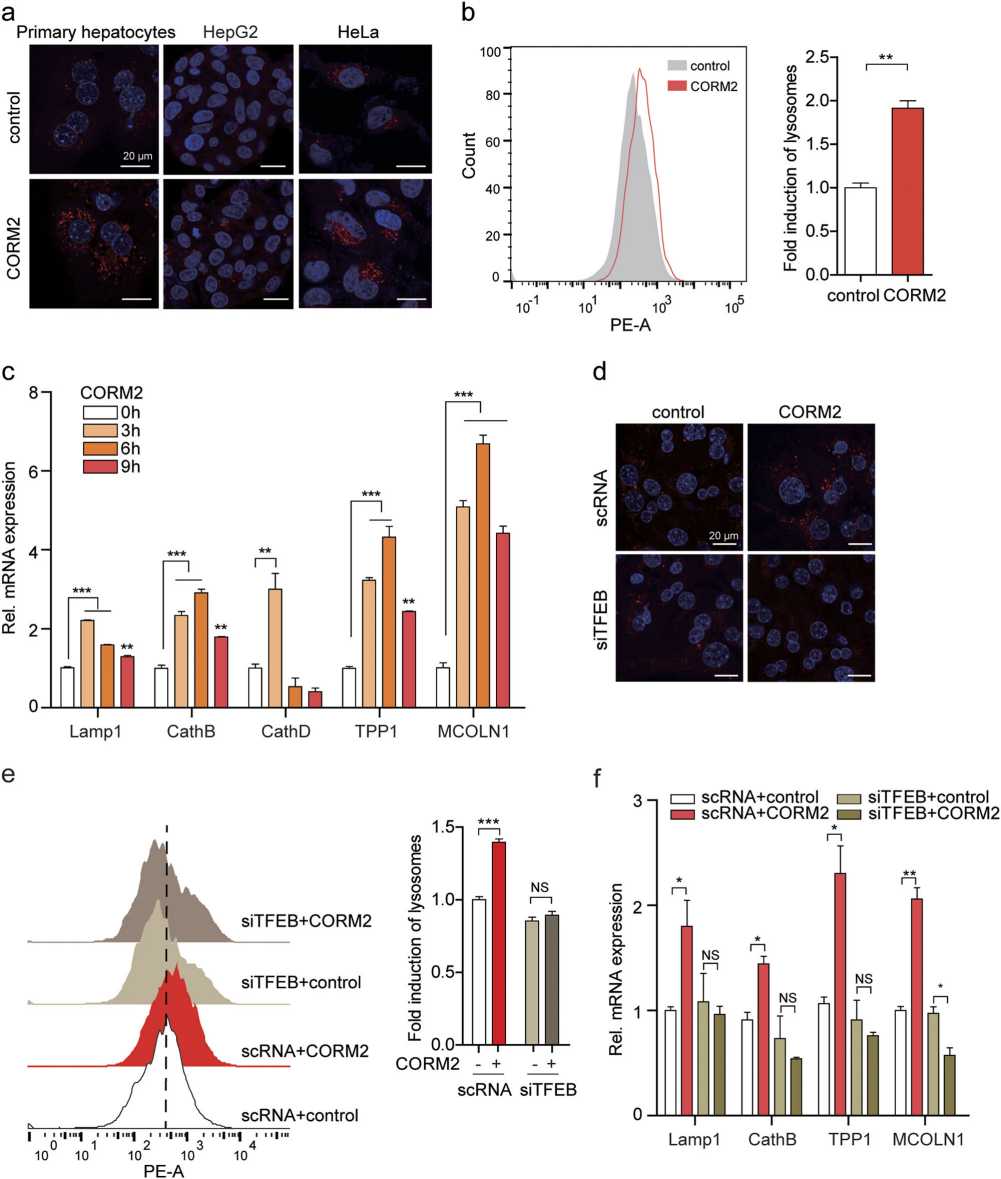
CO promotes lysosomal biogenesis through TFEB activation. **a** Primary hepatocytes, HepG2, and HeLa cells were treated with CORM2 (20 μM) for 6 h and stained with Lysotracker Red staining. Lysosome puncta (red) were captured using a confocal fluorescence microscope. Nuclei were stained with DAPI (blue). Scale bar, 20 μm. **b** Quantification of lysosome (fold induction of LysoTracker staining) of HepG2 cells was assessed by FACS analysis. Data are shown as mean ± SEM (*n* = 3). **P < 0.01 vs. control. **c** Primary hepatocytes were treated with CORM2 (20 μM) for the indicated times. The expression of lysosomal genes, LAMP1, CathB, CathD, TPP1, and MCOLN1 was analyzed by qRT-PCR. Data are shown as mean ± SEM (*n* = 3). **P < 0.01, ***P < 0.001 vs. 0 h. **d**–**f** Primary hepatocytes were transfected with scRNA or siTFEB for 24 h and treated with CORM2 (20 μM) for 6 h. **d** Lysosomes were stained with Lysotracker Red and nuclei were stained with DAPI (blue). Scale bar, 20 μm. **e** Quantifications of lysosome was performed by FACS analysis. **f** The expression of lysosomal genes (LAMP1, CathB, TPP1 and MCOLN1) was analyzed by qRT-PCR. Data are shown as mean ± SEM (*n* = 3). *P < 0.05, **P < 0.01, NS, not significant

Lysosomal biogenesis can be triggered by TFEB, which activates lysosomal and autophagy-related genes, thereby increasing the number of lysosomes and promoting autophagic degradation. We next investigated whether CO induces lysosomal biogenesis through TFEB. CORM2 enhanced lysosome numbers in primary hepatocytes, HepG2, and HeLa cells, as assessed by using LysoTracker Red (Fig. 4a, b). In addition, CORM2 upregulated the expression of lysosome biogenesis-associated genes: lysosomal-associated membrane protein 1 (LAMP1), cathepsin B (CathB), cathepsin D (CathD), tripeptidyl-peptidase 1 (TPP1), and mucolipin 1 (MCOLN1) in primary hepatocytes (Fig. 4c) and in MEF and HepG2 cells (Supplementary Figures S3a, b). The levels of LAMP1 (a lysosomal membrane protein) also were enhanced by CORM2 in MEFs (Supplementary Figure S3c). Knockdown of TFEB significantly reduced lysosome abundance (Fig. 4d, e) and inhibited lysosome biogenesis-associated gene expression (Fig. 4f, Supplementary Figure S3d) in CORM2-treated cells. Next, we examined whether mtROS production and PERK activation were involved in CO-induced autophagy and lysosomal biogenesis. Inhibition of PERK signaling with GSK2606414 decreased CO-induced lysosome abundance in MEFs and CO-induced lysosomal gene expression in primary hepatocytes (Supplementary Figure S3e and S3f). To confirm whether CO-induced lysosomal gene expression is dependent on PERK, we used *Perk*^*+/+*^ and *Perk*^−/−^ MEFs. PERK ablation could not increase the expression of lysosomal genes (Supplementary Figure S3g). Inhibition of mtROS generation with mito-TEMPO decreased CO-induced autophagy (Supplementary Figure S3h) and lysosomal gene expression (Supplementary Figure S3i). Collectively, these results suggested that CO promotes lysosome biogenesis through mtROS, PERK, and activation of TFEB.

### CO induces mitochondrial biogenesis through the PERK-TFEB-PGC1α pathway

**Fig. 5:**
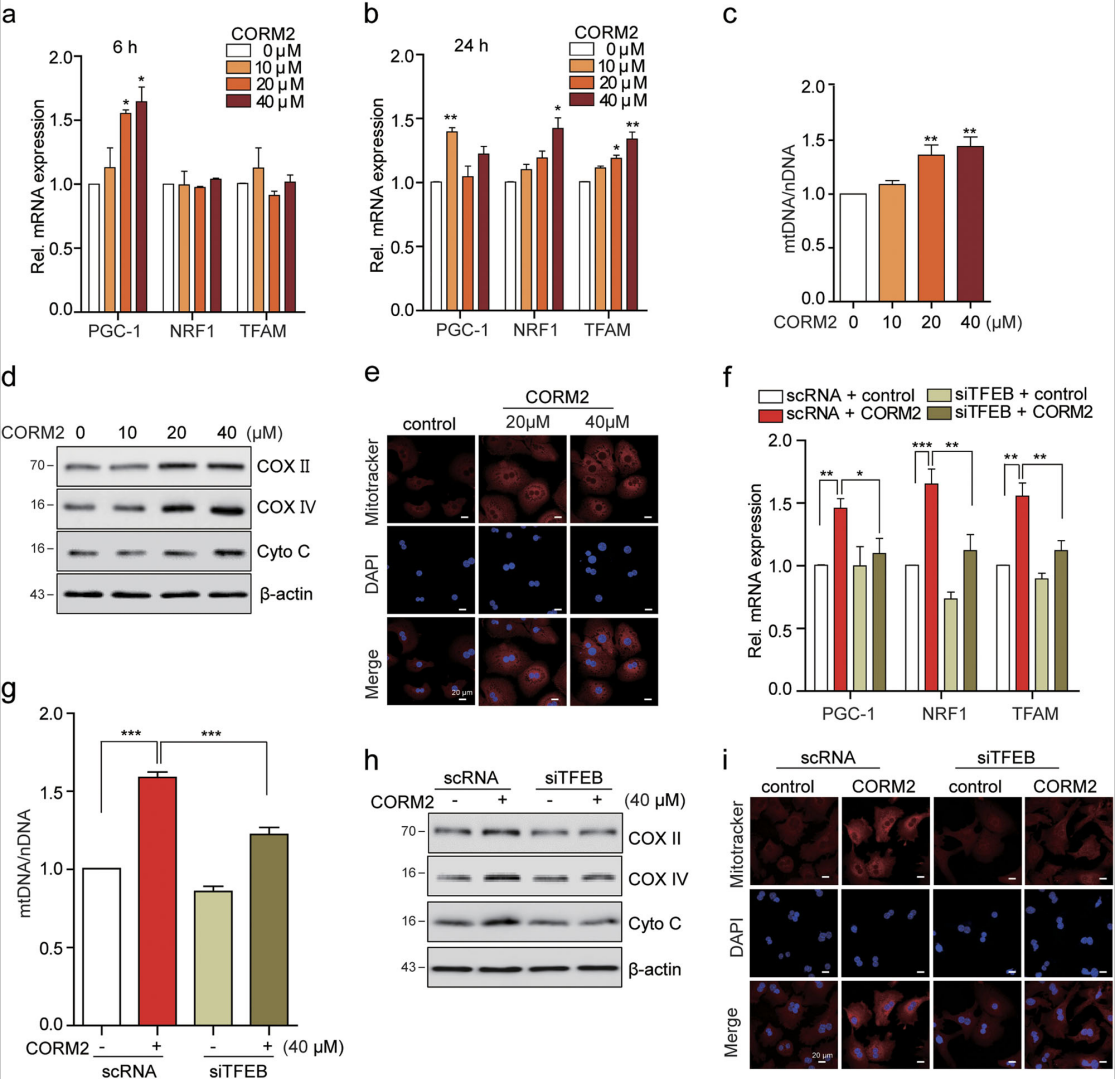
CO induces mitochondrial biogenesis through TFEB-PGC1α pathway. **a**–**e** Primary hepatocytes were treated with CORM2 at the indicated dose for 6 h (**a**) or 24 h (**b–e**). **f**–**i** Primary hepatocytes were transfected with scRNA or siTFEB for 24 h, and then treated with CORM2 for 18 h. **a**, **b**, **f** The expression of PGC1α, NRF1, and TFAM were analyzed by qRT-PCR. Data are shown as mean ± SEM (*n* = 3-4). *P < 0.05, **P < 0.01. **c**, **g** The relative mtDNA content was measured by qRT-PCR. mtDNA content was normalized to nDNA (18s gene) content. Control values were normalized to 1 arbitrary unit. Data are shown as mean ± SEM (*n* = 5–6). **P < 0.01, ***P < 0.001. **d**, **h** The levels of mitochondrial protein, COX II, COX IV, and Cyto c were analyzed by immunoblotting. **e**, **i** Fluorescence intensity of MitoTracker Red (red) and DAPI (blue). Scale bar, 20 μm

TFEB directly regulates PGC1α expression, a master regulator of mitochondrial biogenesis^10^. Previously, we demonstrated that CO activates mitochondrial biogenesis-associated transcriptional coactivators^27^. From the above data, LC3-I/II expression was upregulated early (at 6 h) after the treatment of CO, but was downregulated later (at 24 h). Conversely, TOM20 expression was reduced at 6 h after CO-treating cells, and gradual buildup by 24 h. These results indicate the possibility that following clearance of mitochondria, new mitochondria are synthesized via mitochondrial biogenesis. Given that CO induces the nuclear translocation of TFEB, we investigated whether CO can increase mitochondrial biogenesis through TFEB activation. First, the effect of CORM2 on the expression of genes associated with mitochondrial biogenesis was evaluated in hepatocytes. Treatment with CORM2 significantly increased the levels of PGC1α, nuclear respiratory factor 1 (NRF1), and transcription factor A, mitochondrial (TFAM) mRNA expression in hepatocytes (Fig. 5a, b and Supplementary Figures S4a, b), and we also examined whether CO-induced expression of these genes was dependent on PERK activation. Thus, in the presence of GSK2606414, a PERK inhibitor, the levels of PGC1, TFAM, and NRF1 were not increased with CORM2 treatment (Supplementary Figure S4c). Furthermore, CO increased total mtDNA content in a dose-dependent manner in hepatocytes (Fig. 5c and Supplementary Figure S4d). CO also increased the protein levels of COX II, mitochondria-specific protein COX IV, and Cyto c (Fig. 5d and Supplementary Figure S4e). Furthermore, the increase of mitochondria with CO treatment was examined by confocal microscopy using MitoTracker staining (Fig. 5e and Supplementary Figure S4f). To further examine whether TFEB is required for CO-induced mitochondrial biogenesis, we used scRNA or siTFEB to knock down TFEB gene expression. CO induced the levels of PGC1α, NRF1, and TFAM mRNA expression in primary hepatocytes. The mRNA levels of these factors in hepatocytes transfected with siTFEB were similar to the levels observed in control cells that were not treated with CORM2 (Fig. 5f and Supplementary Figure S4g). In addition, knockdown of TFEB inhibited CO-induced mtDNA contents, levels of COX II, COX IV, and Cyto *c* (Fig. 5g, h and Supplementary Figures S4h, i). To confirm these findings, we performed MitoTracker staining in scRNA or siTFEB-transfected hepatocytes. TFEB knockdown by transfection with siTFEB decreased the abundance of MitoTracker-stained mitochondria after CORM2 treatment (Fig. 5i and Supplementary Figure S4j). These findings suggest that CO-induced mitochondrial biogenesis is mediated by TFEB.

### CO attenuates mitochondrial dysfunction-derived sterile inflammation through TFEB-mediated mitophagy and mitochondrial biogenesis

**Fig. 6:**
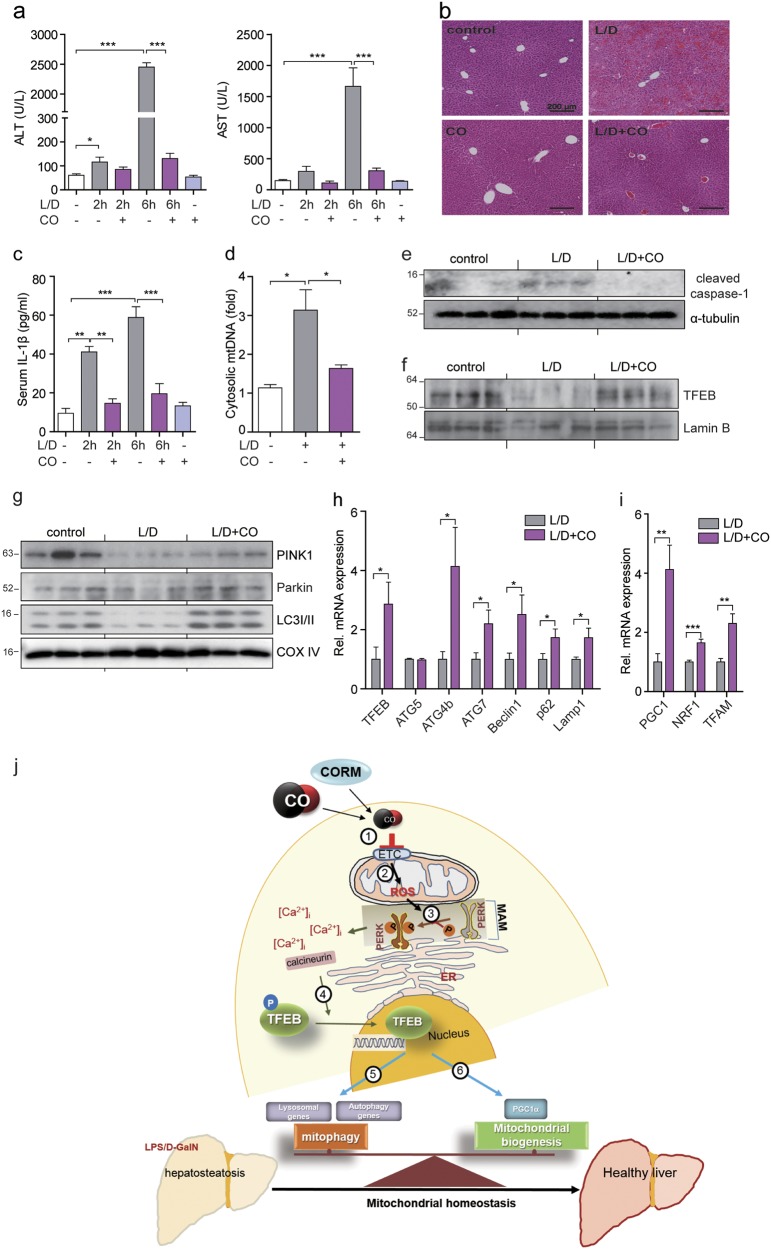
CO attenuates mitochondrial dysfunction-derived sterile inflammation through TFEB-mediated mitophagy and mitochondrial biogenesis. Mice were exposed to LPS/D-galactosamine (D-GalN) with or without CO (250 ppm, 2 h/day) pretreatment, the blood and liver samples were collected 2 h and 6 h (**b**–**i**) after LPS/D-GalN administration. **a** The levels of ALT and AST were determined. Data are shown as mean ± SEM (*n* = 5). ***P < 0.001. **b** Liver sections were stained with H&E for morphological evaluation. **c** The levels of serum IL-1β were measured by ELISA. Data are shown as mean ± SEM (*n* = 4). **P < 0.01. ***P < 0.001. **d** Cytosolic mtDNA amounts in liver tissues were measured by qRT-PCR. Data are shown as mean ± SEM (*n* = 4). *P < 0.05. **e** Cleaved caspase-1 in cell lysates isolated from liver tissues was analyzed by immunoblotting. **f** The level of nuclear TFEB in liver tissues was determined by immunoblotting. Lamin B served as the nuclear standard. **g** Mitochondrial fractionation in liver tissues was analyzed by immunoblotting using antibodies against PINK1, Parkin, and LC3B-II. COX IV was used as marker for mitochondrial fractions. **h**, **i** The levels of autophagy-related genes and lysosomal genes (**h**) or mitochondrial biogenesis-associated genes (**i**) expressed in liver tissues were analyzed by qRT-PCR. Data are shown as mean ± SEM (*n* = 4). *P < 0.05, **P < 0.01. ***P < 0.001. **j** A scheme for the mechanisms of attenuating LSP/D-GalN-induced liver failure by CO through maintaining the balance between mitophagy and mitochondrial biogenesis. CO/CORM inhibits complexes of the mitochondrial respiratory chain (1) and then induces mitochondrial ROS (mtROS) production (2). Increased mtROS leads to PERK activation in the mitochondria-associated membrane (MAM) (3). The phosphorylation of PERK by mtROS increases intracellular calcium concentration. The activation of calcineurin by increased [Ca2 + ]i causes dephosphorylation of TREB (4), followed by its translocation into the nucleus. The nuclear translocation of TFEB increases the expression of genes involved in mitophagy (5) and mitochondrial biogenesis (6)

Accumulation of damaged mitochondria induced by insufficient mitophagy is associated with inflammasome activation^12^. To examine the physiological function of CO, we used mouse model of sterile inflammation. First, we induced fulminant hepatitis with Lipopolysaccharide (LPS) plus D-GalN as acute hepatic inflammatory mouse model. Mice subjected to inhalation of CO displayed attenuated liver damage induced by LPS/D-GalN (Fig. 6a). As shown in Fig. 6b, administration of LPS/D-GalN resulted in hepatocyte vacuolation, blood congestion in the central vein, destruction of the hepatic architecture, and macrophage infiltration. However, CO inhalation prevented the occurrence of pathological changes induced by LPS/D-GalN. Damaged mtDNA, a cellular DAMP, is released into the cytosol during intrinsic apoptosis, and then activates the NLRP3 inflammasome^28,29^. Thus, we hypothesized that CO-attenuated liver damage is associated with reduction in cellular DAMPs released from the mitochondria in response to cellular injury and death caused by LPS/D-GalN. We examined whether CO could reduce mtDNA release and inhibit the LPS/D-GalN-induced sterile inflammatory response. Inhalation of CO significantly reduced mtDNA and IL-1β secretion, and also decreased caspase-1 activation in response to mitochondrial damage (Fig. 6c–e). We next tested whether CO can promote TFEB-mediated mitophagy and mitochondrial biogenesis in vivo to improve LPS/D-GalN-induced liver damage and function. We observed that CO increases the nuclear translocation of TFEB (Fig. 6f), the mitochondrial recruitment of PINK1, Parkin, and LC3 for mitophagy (Fig. 6g), and the expression levels of genes involved in autophagy and lysosome biogenesis (Fig. 6h), or mitochondrial biogenesis (Fig. 6i) in liver tissue. Finally, the scheme (Fig. 6j) depicts the mechanisms by which CO attenuates LSP/D-GalN-induced liver failure through maintaining the balance between mitophagy and mitochondrial biogenesis. These results suggest that CO can decrease the release of DAMPs through elimination of damaged mitochondria and has beneficial effects on LPS/D-GalN-induced liver failure.

## Discussion

In the present study, we demonstrate that CO maintains mitochondrial homeostasis through the regulation of TFEB-mediated mitophagy and mitochondrial biogenesis, processes associated with mitochondrial quality control. The removal of damaged mitochondria by mitophagy, a selective autophagy pathway, and the restoration of normal mitochondrial populations are essential for maintaining cellular homeostasis. Activation of TFEB contributes to sustaining the autophagy-lysosome machinery and enhancing cell survival under various stimuli. Although the roles of TFEB in autophagy and lysosomal biogenesis has been well-clarified^30^, the identification of molecules that can regulate TFEB remain largely unknown. Previously, our study showed that CO activates PERK signaling through generation of mtROS^31^. In this study, we show that a mild increase in mtROS levels in response to CO treatment leads to PERK activation, Ca^2+^ release, and calcineurin-dependent TFEB nuclear translocation (Figs. 1, 2), which in turn triggers the activation of autophagy and lysosomal biogenesis (Figs. 3, 4). In addition, activation of TFEB by CO facilitates mitophagy through enhanced Parkin recruitment to the mitochondria (Fig. 3) and induces mitochondrial turnover through the upregulation of PGC1α to stimulate mitochondrial biogenesis (Fig. 5), which restores the population of normal mitochondria. Moreover, mtROS can contribute to the opening of the mitochondrial permeability transition pore (mPTP), which can induce mitophagy^32^. Cyclophilin D (CypD) is an essential component of mPTP and the mPTP is blocked by CsA, which binds to CypD^33^. Our results demonstrate that both CsA and FK506 inhibit CORM2-induced nuclear translocation of TFEB (Fig. 2e and Supplementary Figure S1h). It is known that CsA, but not FK506, inhibits the opening of mPTP^34^, and that CO induces Parkin recruitment to the mitochondria through TFEB, promoting mitophagy (Fig. 3). This suggests that mitophagy is induced via TFEB activation, although further research is necessary to elucidate the causal relationship between mPTP and mitophagy.

The impairment of mitochondrial quality control processes may lead to the accumulation of intracellular oxidized molecules and their release as DAMPs. Damaged mitochondria can trigger cellular necrosis, with the release of nDNA fragments, mtDNA, and proteins such as high-mobility group box 1 that cause transcriptional activation of pro-inflammatory cytokines through TLRs binding and inflammasome activation in local inflammatory cells in the liver^35^. DAMPs are released from the mitochondria in response to cell damage and death can induce sterile inflammation^12,36^. Acetaminophen-induced protein adduct formation has been shown to trigger mitochondrial damage, which can lead to necrotic cell death and subsequent liver injury^37^. Acute hepatic inflammation induced by LPS/D-GalN was inhibited by celastrol-induced mitophagy^38^. As mitochondrial damage is sufficient to augment tissue injury, removal of damaged mitochondria to maintain normal mitochondrial function may be necessary for protection against tissue injury. According to a previous report, myocardial injury resulted in impaired TFEB activation^4^. Therefore, activating TFEB is likely an efficient regulator to eliminate dysfunctional mitochondria induced by various cellular stress conditions.

Exogenous CO inhalation has been shown to protect mice from cardiomyopathy by modulating signaling through the Akt1 pathway which is dependent on mitochondrial biogenesis^39^. Previously, our reports have demonstrated that CO activates mitochondrial biogenesis via inducing transcriptional coactivators^27^. In addition, overexpression or knockout of the HO-1 gene affected mitochondrial quality control through changes of PINK1 and Park2 (Parkin) gene expression. HO-1 overexpression has shown to protect against doxorubicin-induced cardiomyopathy through induction of the PINK1 and Parkin expression, whereas HO-1 deficiency resulted in failure to upregulate PINK1 and Parkin-mediated mitophagy^40,41^. Consistent with above reports, we observed that CO upregulates mitochondrial PINK1 and Parkin, and gene expression associated with mitochondrial biogenesis, which might confer beneficial properties in hepatocytes (Figs. 3–5).

Expression of the key mediators of mitophagy, PINK1 and Parkin, were decreased at early time points in mice treated with doxorubicin^40^. In addition, PINK1 protein levels are markedly reduced in end-stage human heart failure. Another study has shown that Parkin expression was decreased by LPS and tumor necrosis factor in an nuclear factor-κB-dependent manner^42^. Our study demonstrated that LPS/D-GalN inhibits mitophagy in control mice, whereas activation of mitophagy is preserved in LPS/D-GalN-treated mice subjected to CO inhalation. LPS/D-GalN appeared to inhibit the levels of mitochondrial PINK1 compared with control mice (Fig. 6). The inhibition of PINK1 appears to correlate with upregulation of ER stress, which contributes to liver inflammation and hepatotoxicity in acute liver failure. Transcriptional repression of PINK1 was mediated by ER stress^43^. The suppression of mitophagy may worsen LPS/D-GalN-induced liver injury by impairing the clearance of damaged mitochondria. Here we observed that inhalation of CO prevents LPS/D-GalN-induced liver injury by activating TFEB. During acute liver failure induced by LPS/D-GalN, CO induced the levels of mitochondrial PINK1 and Parkin, and PGC1α-mediated NRF1 and TFAM gene expressions, which could lead to promotion of mitochondrial turnover and biogenesis. These events may lead to prevent sterile inflammation triggered by the presence of damaged mitochondria. Finally, the protective effect of CO against LPS/D-GalN-induced acute hepatic inflammation in mice is likely related to mitochondrial quality control which is dependent on both TFEB-mediated mitophagy and mitochondrial biogenesis (Fig. 6). These observations suggest that CO-induced TFEB activation has a role in removing dysfunctional mitochondria. A limitation of our study is that the mechanistic and genetic validation studies were performed largely in vitro using cultured hepatocytes that were not directly exposed to injury. Our in vivo studies demonstrating CO-dependent protection in a hepatic injury model however suggest association with downregulation of ER stress and upregulation of mitophagy and the expression of mitophagy-related factors.

In summary, our data indicate that TFEB activation by CO regulates the autophagic turnover of damaged mitochondria and their subsequent replacement through mitochondrial biogenesis. CO has a protective role in acute liver failure as it promotes the clearance and recycling of damaged mitochondria from cells affected by liver tissue injury. These results suggest that CO therapy may represent a potentially effective strategy to prevent diseases associated with mitochondrial dysfunction.

## Materials and methods

### Reagents

Tricarbonyldichlororuthenium (II) dimer (CORM2), PERK inhibitor (GSK2606414), (2-(2,2,6,6-Tetramethylpiperidin-1-oxyl-4-ylamino)-2-oxoethyl)triphenylphosphonium chloride (Mito-TEMPO), Rotenone, Cyclosporin A, FK506, LPS (*Escherichia coli* O111:B4), and d-galactosamine hydrochloride (D-GalN) bafilomycin A1 were purchased from Sigma-Aldrich (St. Louis, MO, USA). Torin 1 (4247) and ryanodine were purchased from Tocris Bioscience.

### Cell culture

HepG2, primary hepatocytes, and HeLa cells were maintained in Dulbecco’s modified Eagle’s medium (DMEM; GIBCO, Grand Island, NY) supplemented with 10% heat-inactivated fetal bovine serum (FBS) and 1% penicillin/streptomycin (P/S) (GIBCO). AML12 cells were cultured in DMEM/F12 supplemented with 10% FBS and 1% P/S. *Perk*^+/+^ mouse embryonic fibroblasts (MEFs) and *Perk*^−/−^ MEFs were maintained in DMEM medium supplemented with 10% FBS, 1% P/S, and MEM non-essential amino acid solution (GIBCO 11140-050). All cells were incubated at 37 °C with 100% humidity in 5% CO_2_ and passaged using standard cell culture techniques. For starvation, cells were washed three times in Hanks’ Balanced Salt Solution (HBSS) with 10 mM HEPES for 3 h at 37 °C.

### Animals and treatment

All experiments with mice were approved by the Animal Care Committee of the University of Ulsan. C57BL/6 mice (6 weeks old, male) were purchased from ORIENT (Busan, Korea). For the fulminant hepatitis mouse model, mice (10 weeks old, male) were injected with LPS (0.25 μg/mouse, inraperitoneally (i.p.)) and D-GalN hydrochloride (15 mg/mouse, i.p.). Mice (*N* = 5) were subjected to inhalation of air or CO at 250 parts per million (p.p.m.) in air (Core Gas Ulsan, Korea) for 7 days (2 h/day) before challenge with LPS and D-GalN in a sealed exposure chamber (LB Science, Daejeon, South Korea). Mice were killed at 2 h and 6 h after LPS and D-GalN administration, and then serum and liver tissues were collected for analyses.

### Isolation of primary hepatocytes

Primary hepatocytes were isolated from mice by collagenase digestion and by the two-step Percoll gradient method with slight modifications. Mice were anesthetized and the peritoneal cavity was opened. Livers were perfused with Ca^2+^ and Mg^2+^-free Hanks' Balanced Salt Solution (HBSS) (GIBCO) containing EGTA (2.5 mM) and then digested with a collagenase buffer containing collagenase (0.5 mg/ml, C5138, Sigma), NaCl (66.7 mM), KCl (6.7 mM), HEPES (50 mM), and CaCl_2_ (4.8 mM). Digested livers were dissected and then gently teased with forceps until they were in solution. The cell suspensions were filtered through a 100 μm nylon cell strainer (BD Falcon, CA, USA). The cells were centrifuged for 5 min at 1900 r.p.m. and resuspended in Hanks' Balanced Salt Solution (HBSS). After the cell suspensions were centrifuged for 3 min at 450 r.p.m., the pellets (parenchymal cells such as hepatocytes) were resuspended with Hanks' Balanced Salt Solution (HBSS). The pellet suspensions obtained as described above were centrifuged using 25% Percoll for 5 min at 800 r.p.m. with the brake option off. The pellets were washed with DMEM supplemented with 10% FBS and then cells were seeded into a collagen pre-coated 100 mm tissue culture plates. After 24 h, non-adherent cells were removed by aspiration and fresh media were added.

### Hepatocellular damage assay

To detect serum alanine aminotransferase (ALT) and aspartate aminotransferase (AST), serum was collected from peripheral blood. ALT and AST activity, indicators of hepatocellular injury, were measured using the EnzyChrom™ Alanine Transaminase Assay Kit and EnzyChrom™ Aspartate Transaminase Assay Kit (BioAssay System, Hayward, CA).

### Mitochondrial ROS measurement by FACS

Cells were grown to 80% confluence and fresh media was replaced before the experiments. Cells were treated with the CO-releasing molecule CORM2 (20 μM) for 6 h in the absence or presence of Mito-TEMPO (100 μM). After treatment, MitoSOX Red (2 μM) was added and the cells were incubated in the dark for 30 min at 37 °C. The cells were trypsinized and were placed in fluorescence-activated cell sorting (FACS) tube. Subsequently, cells were washed three times with phosphate-buffered saline (PBS) and then samples were diluted to a final volume of 500 μl with FACS buffer. MitoSOX fluorescence was detected by FACSCanto™ flow cytometry system (BD Biosciences). Data were analyzed using FlowJo software (version 10).

### Plasmid, siRNA transfection, and luciferase assay

mCherry-EGFP-LC3B, pEGFP-N1-TFEB, and 4X CLEAR-luciferase reporter were purchased from Addgene. RFP-LC3 was obtained from Sung Hoon Back (University of Ulsan, Korea). pEGFP-N1 vector was used to insert the mitochondria targeting sequence derived from human cytochrome c (Cyto c) oxidase subunit 8a (Cox8a). Cells were transfected with pEGFP-TFEB or 4X CLEAR-luciferase reporter construct and pRL-SV40 Renilla luciferase construct (Promega) using Lipofectamine™ 2000 (Invitrogen, Carlsbad, CA) in accordance with the manufacturer’s protocol. Cells were transfected with TFEB small interfering RNA (siRNA) (h) (sc-38509, Santa Cruz Biotechnology, Santa Cruz, CA), TFEB siRNA (m) (sc-38510, Santa Cruz Biotechnology), or scramble siRNA (scRNA) (AM4611, Ambion, Austin, TX) using Lipofectamine™ RNAiMAX reagent (Invitrogen, Carlsbad, CA). After 24–48 h, plasmids or siRNA-transfected cells were assayed fluorescence microscopy, quantitative reverse-transcriptase PCR (qRT-PCR), or immunoblotting. For luciferase assay, cells were co-transfected with 4X CLEAR firefly luciferase reporter construct and pRL-SV40 *Renilla* luciferase construct, and lysed with lysis buffer and mixed with luciferase assay reagent (Promega), and the chemiluminescent signal was measured in a SpectraMax L Microplate (Molecular Devices, Sunnyvale, CA). Firefly luciferase was normalized to *Renilla* luciferase in each sample. All luciferase assays reported here represent at least three independent experiments, each consisting of three wells per transfection.

### Quantitative real-time PCR

Total RNA was isolated from cells using Trizol (Life Technologies) according to the manufacturer’s protocol. RNA was reverse transcribed to synthesize the first-strand cDNA by using oligo (dT) primers (Bioneer, Daejeon, Korea) and M-MLV reverse transcriptase (Promega) according to the manufacturer’s instructions. The cDNA product was subjected to the PCR-based amplification. Real-time PCR was performed using SYBR Green PCR Master Mix (Applied Biosystems, Foster City, CA) on an ABI 7500 Fast Real-Time PCR System (Applied Biosystems). Real-time PCR primer pairs are listed in Supplementary Table 1. mRNA expression data were normalized to GAPDH gene expression.

### Immunoblotting

Collected tissues and cells were lysed with mammalian lysis buffer or RIPA buffer containing phosphatase and protease inhibitors. Proteins were resolved in SDS-polyacrylamide gel electrophoresis gel and transferred to polyvinylidene difluoride membranes (GE Healthcare, Waukesha, WI). The membranes were blocked for 1 h in PBS-Tween 20 (PBS-T) containing 5% skim milk and incubated with appropriate dilutions of antibodies at 4 °C overnight as follows: phospho-PERK (#649401, BioLegend; MA5-15033, Thermo Scientific; #12814, Signalway Antibody SAB), TFEB (#4240, Cell Signaling; A303-673A, Bethyl Laboratories), GFP (sc-9996, Santa Cruz Biotechnology), TOM20 (sc11415, Santa Cruz Biotechnology), LC3 (NB100-2220, Novus Biologicals, Littleon, CO), PINK1 (BC100-494, Novus Biologicals), Parkin (ab15954, Abcam), LAMP1 (ab24170, Abcam), complex II (COX II) (sc-1746, Santa Cruz Biotechnology), Cyto c oxidase subunit IV (COX IV) (#4844 S, Cell Signaling), Cyto c (556433, BD Bioscience), α-tubulin (#2125, Cell Signaling), caspase-1 (sc-56036, Santa Cruz Biotechnology), PARP (#9532, Cell Signaling), Lamin B (sc-6216, Santa Cruz Biotechnology), and β-actin (#4967 S, Cell Signaling) antibodies were used. α-Tubulin and β-actin served as the standard. Membranes were then washed with 0.05% PBS-T and incubated with a 1/5000 dilution of horseradish peroxidase-conjugated secondary antibodies at room temperature for 1 h. Immunoreactivity was detected using the ECL detection system (GE Healthcare) and chemiluminescence signal was read with an Azure Biosystems C300 (Azure Biosystems, Inc., Dublin, CA).

### Subcellular fractionation

The nuclear fraction was extracted from cells using nuclear/cytosol fractionation kit (K266, Biovision, Mountain View, CA, USA). Cells were collected and subcellular fractionation was performed according to the manufacturer’s instructions. Briefly, cells were collected in PBS and centrifuged at 4 °C for 5 min at 5000 × *g* in a microcentrifuge. Cell pellets were resuspended in cytosolic extraction buffer A (CEB-A) and incubated for 10 min on ice before addition of CEB-B. After 1 min vortexing, the lysates were centrifuged at 4 °C for 5 min at 16,000 × *g* in a microcentrifuge and the supernatants were kept as the cytoplasmic fractions. The nuclear pellet was resuspended in nuclear extraction buffer and vortexed for 20 s. This step was repeated every 10 min five times. The nuclear pellet was centrifuged at 4 °C for 10 min at 16,000 × *g*, and the supernatant was kept as a nuclear fraction. The nuclear fractions were analyzed by immunoblotting using antibodies against α-tubulin as a cytosolic marker and Lamin B or PARP1 as a nuclear marker.

### Mitochondrial DNA analysis

Total DNA was extracted from primary hepatocytes and HepG2 cells using a Blood and Cell Culture DNA Mini Kit (Qiagen). Mitochondrial DNA (mtDNA) copy number was measured by real-time PCR. The following primers for mtDNA were used: cytochorome c oxidase subunit I: forward primer 5′-CAAACCTACGCCAAAATCCA-3′, reverse primer 5′-GAAATGAATGAGCCTACAGA-3′. Mouse cytochrome b (*Mus musculus* domesticus mitochondrion): forward primer 5′-CCACTTCATCTTACCATTTA-3′, reverse primer 5′-ATCTGCATCTGAGTTTAATC-3′. The following primers for nuclear DNA (nDNA) were used: human β-actin: forward primer 5′-TCACCCACACTGTGCCCATCTACGA-3′, reverse primer 5′-CAGCGGAACCGCTCATTGCCAATGG-3′; and mouse 18S rRNA: forward primer 5′-GGGAGCCTGAGAAACGGC-3′, reverse primer 5′-GGGTCGGGAGTGGGTAATTT-3′. Relative amounts of mtDNA and nDNA copy numbers were compared.

### Mitochondrial extraction and measurement of cytosolic mtDNA

For mitochondria, extraction was performed by mitochondria isolation kit (#89874, Thermo Fisher, lL, USA). Cells were collected and resuspended in buffer A. Then, mitochondria isolation reagent B added and incubated on ice for 5 min, added Reagent C, and centrifuged at 700 × *g* for 10 min at 4 °C. Supernatant were transferred to fresh tube and then centrifuged at 12,000 × *g* for 15 min at 4 °C. Supernatant and pellet contained cytosolic fraction/mitochondria fraction, respectively. Mitochondrial fractionation from liver tissue was performed using a mitochondrial isolation kit (#89801, Thermo Fisher, lL, USA) according to manufacturer’s instructions. Measurement of cytosolic mtDNA was performed by using the mitochondrial isolation kit above. Protein concentration and volume of the cytosol extracts were normalized. DNA was isolated from 300 µl of the cytosolic fractions and mtDNA encoding cytochrome b was measured by quantitative real-time PCR with same volume of the DNA solution.

### Fluorescence microscopy

Cells were plated on a four-well Lab-Tek chambered coverglass (Nunc, Thermo Scientific, Waltham, MA) and transfected with pEGFP-TFEB plasmid. After 24 h from transient transfection, cells were treated with 20 μM CORM2 for 3 h. GFP-TFEB location was imaged with an Olympus FV1200 confocal microscopy (Olympus, Tokyo, Japan). For detection of autophagosomes and autolysosomes, cells were transfected with the expression vector mcherry-GFP-LC3 for 24 h. To detect mitophagosome, cells were co-transfected with GFP-mitoTS and RFP-LC3 plasmids for 24 h. After transfecting, cells were treated with CORM2. For Lysosome staining, cells were cultured in medium containing LysoTracker Red (L7528, Thermo Scientific) (0.3 μM) for 30 min. After washing in PBS, cells were fixed with 4% paraformaldehyde in PBS at room temperature for 10 min and the washed with PBS, and then stained with 1 μg/ml 4′,6-diamidino-2-phenylindole (DAPI) (Sigma, D9542) for 20 min. The samples were washed with PBS. Lysosome was imaged with an Olympus FV1200 confocal microscopy (Olympus, Tokyo, Japan).

To assess changes in mitochondrial mass, mitochondrial staining was performed by incubating cells with MitoTracker Red CMXRos (Invitrogen). After experimental treatment, cells were incubated with MitoTracker Red CMXRos (500 nM) for 30 min at 37 °C in dark, then subsequently washed twice in PBS and fixed with 4% paraformaldehyde in PBS at 37 °C for 30 min. Cells were analyzed by using an FV1000 Confocal Laser Scanning Biological Microscope at excitation wavelength 579 nm and emission wavelength 599 nm.

### Hematoxylin and eosin staining

For histopathological observations, portions of liver were fixed in 10% neutral-buffered formalin solution and then dehydrated in graded alcohol. The fixed tissue was embedded in paraffin and sliced into 4 μm-thick sections. Tissue sections were mounted on regular glass slides, deparaffinized in xylene, rehydrated in decreasing concentrations of ethanol, and stained with hematoxylin and eosin.

### Measurement of intracellular Ca^2+^ concentration

Hepatocytes or MEF cells seeded on collagen-coated confocal dishes were incubated with 5 μM fluo-4 AM (Invitrogen) in Medium 199 (GIBCO) containing 1% bovine serum albumin at 37 °C for 40 min as described previously^44^. The cells were washed three times with Medium 199. Changes of intracellular Ca^2+^ concentration ([Ca^2+^]_i_) were determined at 488 nm excitation/530 nm emission using an air-cooled argon laser system. The emitted fluorescence at 530 nm was collected using a photomultiplier. The image was scanned using a confocal microscope (Nikon, Japan). For the calculation of [Ca^2+^]i, the method of Tsien et al.^45^ was used with the following equation: [Ca^2+^]i = *K*_d_(*F* − *F*_min_)/(*F*_max_ − *F*), where *K*_d_ is 345 nM for fluo-4 and *F* is the observed fluorescence level. Each tracing was calibrated for the maximal intensity (*F*_max_) by addition of ionomycin (8 μM) and for the minimal intensity (*F*_min_) by addition of EGTA 50 mM at the end of each measurement.

### Enzyme-linked immunosorbent assay

Serum and tissue homogenate were analyzed for cytokine levels using commercially available mouse IL-1β DuoSet ELISA kits (R&D Systems, Minneapolis, MN, USA), according to the manufacturer’s instructions.

### Statistical analysis

All data were represented as mean ± SD or SEM. The data were analyzed using GraphPad Prism 5 (GraphPad Software, Inc., La Jolla, CA, USA). Statistical differences were assessed with one-way analysis of variance (ANOVA) followed by the Tukey’s multiple comparison post hoc test or the Kruskal–Willis for a non-parametric test, and two-way ANOVA with Bonferroni’s post test or Mann–Whitney test. *P* < 0.05 was considered significant.

## Electronic supplementary material

Carbon Monoxide-Induced TFEB Nuclear Translocation Enhances Mitophagy/Mitochondrial Biogenesis in Hepatocytes and Ameliorates Inflammatory Liver Injury
