# Supplementary material for: Carbon monoxide-induced TFEB nuclear translocation enhances mitophagy/mitochondrial biogenesis in hepatocytes and ameliorates inflammatory liver injury
Source: Cell Death Dis. 2018 Oct 17;9(11):1060. doi: 10.1038/s41419-018-1112-x (PMC6193007; doi:10.1038/s41419-018-1112-x)
Supplement: Supplementary file 1 — Carbon Monoxide-Induced TFEB Nuclear Translocation Enhances Mitophagy/Mitochondrial Biogenesis in Hepatocytes and Ameliorates Inflammatory Liver Injury [file 41419_2018_1112_MOESM1_ESM.docx]

**Supplementary Information**

**Carbon Monoxide-Induced TFEB Nuclear Translocation Enhances Mitophagy/Mitochondrial Biogenesis in Hepatocytes and Ameliorates Inflammatory Liver Injury**

Hyo Jeong Kim^1^, Yeonsoo Joe^1^, So-Young Rah^2^, Seul-Ki Kim^1^, Se-Ung Park^1^,

Jeongmin Park^1^, Jin Kim^1^, Jinhyun Ryu^3^, Gyeong Jae Cho^3^, Young-Joon Surh^4^,

Stefan W. Ryter^5^, Uh-Hyun Kim^2^*, Hun-Taeg Chung^1^*

^1^Department of Biological Sciences, University of Ulsan, Ulsan 680-749, Republic of Korea.

^2^National Creative Research Laboratory for Ca2+ Signaling Network, Chonbuk National University Medical School, Jeonju, Republic of Korea. ^3^Department of Anatomy, School of Medicine and Institute of Health Sciences, Gyeongsang National University, JinJu 660-701, Korea. ^4^Tumor Microenvironment Global Core Research Center and Research Institute of Pharmaceutical Sciences, College of Pharmacy, Seoul National University, Seoul, Republic of Korea. ^5^Joan and Sanford I. Weill Department of Medicine, Division of Pulmonary and Critical Care Medicine, Weill Cornell Medical Center, New York, NY, USA.

**Address correspondence to:Uh-Hyun Kim,** [**uhkim@chonbuk.ac.kr**](mailto:uhkim@chonbuk.ac.kr)**; Hun Taeg Chung,** [**chung@ulsan.ac.kr**](mailto:chung@ulsan.ac.kr)

**Uh-Hyun Kim Hun-Taeg Chung**

Medical School, Chonbuk National University School of Biological Sciences,

Jeonju, Republic of Korea University of Ulsan, Ulsan Korea

<Tel:+82-63-270-3083> Tel: +82-52-259-2392

Fax:+82-63-274-9833 Fax: +82-52-259-2740

E-mail:uhkim@chonbuk.ac.kr E-mail: [chung@ulsan.ac.kr](mailto:chung@ulsan.ac.kr)

**Supplementary Table 1.** Primer sequences used for quantitative RT-PCR

| **Primer** | **Sequence (5’→3’)** |
| --- | --- |
| mQ-MCOLN1-F | gcgcctatgacaccatcaa |
| mQ-MCOLN1-R | tatcctggactgctcgat |
| mQ-TPP1-F | aagccaggcctacatagtcaga |
| mQ-TPP1-R | ccaagtgcttcctgcagtttaga |
| mQ-CathB-F | ttagcgctctcacttccactacc |
| mQ-CathB-R | tgcttgctaccttcctctggtta |
| mQ-Lamp1-F | TAATGGCCAGCTTCTCTGCCTCCTT |
| mQ-Lamp1-R | AGGCTGGGGTCAGAAACATTTTCTT |
| mQ-CathD-F | AACTGCTGGACATCGCTTGCT |
| mQ-CathD-R | CATTCTTCACGTAGGTGCTGGA |
| mQ-GAPDH-F | GGGAAGCCCATC ACCATCT |
| mQ-GAPDH-R | CGGCCTCACCCCATTTG |
| hQ-MCOLN1-F | gagtgggtgcgacaagtttc |
| hQ-MCOLN1-R | tgttctcttcccgga atgtc |
| hQ-TPP1-F | gatcccagctctcctcaatac |
| hQ-TPP1-R | gccatttttgcaccgtgtg |
| hQ-CathD-F | cttcgacaacctgatgcagc |
| hQ-CathD-R | tacttggagtctgtgccacc |
| hQ-CathB-F | agtggagaatggcacacccta |
| hQ-CathB-R | aagagccattgtcacccca |
| hQ-LAMP1-F | CGTACCTTTCCAACAGCAGC |
| hQ-LAMP1-R | CGCTCACGTTGTACTTGTCC |
| hQ-GAPDH-F | CAATGACCCCTTCATCCTC |
| hQ-GAPDH-R | AGCATCGCCCCACTTGATT |
| hQ-PGC1α-F | TGAGAGGGCCAAGCAAAG |
| hQ-PGC1α-R | ATAAATCACACGGCGCTCTT |
| mQ-PGC1α-F | AGCCGTGACCACTGACAACGAG |
| mQ-PGC1α-R | GCTGCATGGTTCTGAGTGCTAAG |
| hQ-NRF1-F | CCATCTGGTGGCCTGAAG |
| hQ-NRF1-R | GTGCCTGGGTCCATGAAA |
| mQ-NRF1-F | CGCAGCACCTTTGGAGAA |
| mQ-NRF1-R | CCCGACCTGTGGAATACTTG |
| hQ-TFAM-F | GAACAACTACCCATATTTAAAGCTCA |
| hQ-TFAM-R | GAACAACTACCCATATTTAAAGCTCA |
| mQ-TFAM-F | GGAATGTGGAGCGTGCTAAAA |
| mQ-TFAM-R | TGCTGGAAAAACACTTCGGAATA |
| mQ-TFEB-F | gcgagagctaacagatgctga |
| mQ-TFEB-F | ccggtcattgatgttgaacc |
| mQ-ATG5-F | GAATATTTTGAATCTCCCGTTCC |
| mQ-ATG5-R | GGCCACAATGACATAATCCA |
| mQ-ATG4b-F | GGGAACTGGCCCTACTTCAGA |
| mQ-ATG4b-R | TCCACCTCCAATCTCGACCTA |
| mQ-ATG7-F | CAGTTTCCAGTCCGTTGAAGTCCT |
| mQ-ATG7-R | GGGTCCATACATCCACTGAGGTTC |
| mQ-Beclin1-F | TGAAATCAATGCTGCCTGGG |
| mQ-Beclin1-R | CCAGAACAGTATAACGGCAACTCC |
| mQ-p62-F | TGTGGAACATGGAGGGAAGAG |
| mQ-p62-R | TGTGCCTGTGCTGGAACTTTC |

**
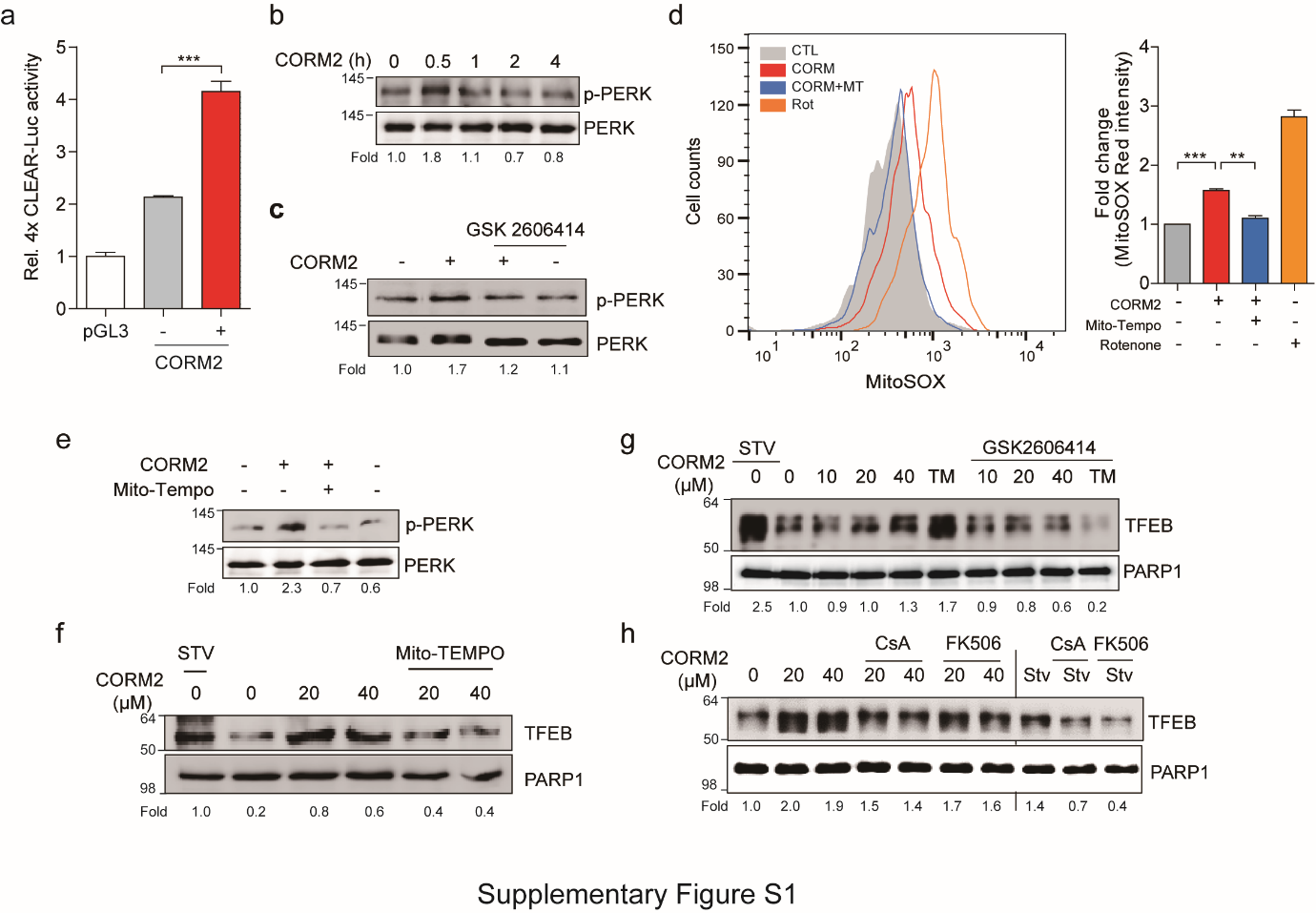
**

**Supplementary Fig. S1. Carbon monoxide (CO) increases TFEB nuclear translocation in HepG2 cells. (a)** HepG2 cells were co-transfected with 4X CLEAR-luciferase reporter construct and pRL-SV40 renilla luciferase construct for 24 hours. After treatment with CORM2 for 9 hours, cells were lysed and assayed for luciferase activity. Values are shown as mean ± SD (n=3). Data are shown as mean ± SEM. ****P*<0.001. **(b)** HepG2 cells were treated with CORM2 (20 μM) for the indicated times. The levels of phosphorylated PERK (p-PERK) and total PERK were measured by immunoblotting. **(c)** HepG2 cells were pretreated with GSK2606414 (PERK inhibitor, 0.5 μM) for 30 min, and then treated with CORM2 (20 μM) for 1 hour. The levels of phosphorylated PERK (p-PERK) and total PERK were measured by immunoblotting. **(d)** Primary hepatocytes were treated with CORM2 (20 μM) for 6 h in the absence or presence of Mito-TEMPO (100 μM) with MitoSOX Red and analyzed by flow cytometry. Rotenone was used as a positive control for mitochondrial ROS production. Data are shown as mean ± SEM. ***P<0.001. **(e)** Primary hepatocytes were treated with CORM2 (20 μM) for 1 h in the absence or presence of Mito-TEMPO (100 μM). After treatment, cells were lysed and analyzed by immunoblotting using antibodies against phosphorylated (p)-PERK and PERK. **(f)** Primary hepatocytes were treated with CORM2 (20 μM) for 1 h or starved for 3 h in the absence or presence of Mito-TEMPO (100 μM), and then prepared nuclear extracts. Endogenous nuclear TFEB was analyzed by immunoblotting. **(g)** HepG2 cells were pretreated with GSK2606414 for 30 min, and then cells were treated with CORM2 or tunicamycin (TM). Endogenous nuclear TFEB was analyzed by immunoblotting. **(h)** HepG2 cells were incubated with CORM2 or starved for 3 hours in the presence or absence of calcineurin inhibitor, cyclosporin A (10 μM) or FK506 (5 μM) for 1 hour. PARP1 served as the nuclear standard. Band intensities were determined by densitometry (Image J). Basal levels in the untreated sample were set at 1.0, and results were expressed as fold induction over control levels.


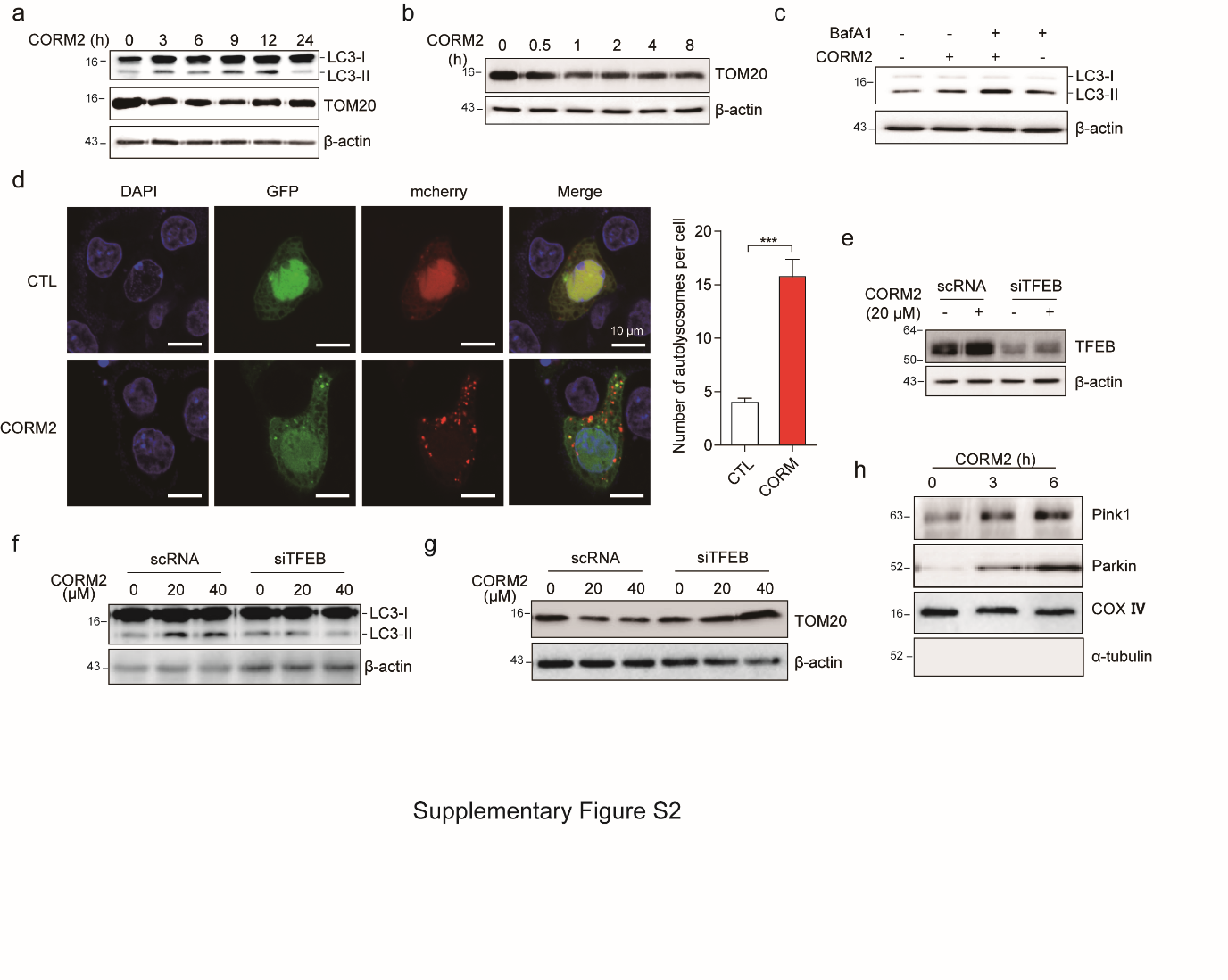


**Supplementary Fig. S2. CO enhances mitophagy in HepG2 cells.** **(a, b)** HepG2 cells were treated with CORM2 (20 μM) for the indicated times. The levels of LC3B-II and TOM20 were determined by immunoblotting. (**c**) HepG2 cells were treated with CORM2 (20 μM) in the presence of Bafilomycin A1 (Baf A1), autophagy flux inhibitor. The cells were harvested and cell lysates were subjected to immunoblotting to detect the expression of LC3B. (**d**) HepG2 cells were transfected with mcherry-GFP-LC3 for 24 hours. After transfection, cells were treated with CORM2 (20 μM) for 6 hours, and analyzed by confocal fluorescence microscopy. Scale bar, 10 μm. The number of autolysosomes (red puncta) per cell (n=10) was counted. Data are shown as mean ± SEM. ****P*< 0.001. (**e**) Mitochondrial fractions isolated from HepG2 cells treated with CORM2 (20 μM) for the indicated times were analyzed by immunoblotting using antibodies against PINK1 and Parkin. (**f-h**) HepG2 cells were transfected with scrambled RNA (scRNA) or siRNA against TFEB (siTFEB) for 24 hours, and then treated with CORM2 at the indicated doses for 6 hours. The levels of TFEB (**f**), LC3B-II (**g**) and TOM20 (**h**) were assessed by immunoblotting.

**
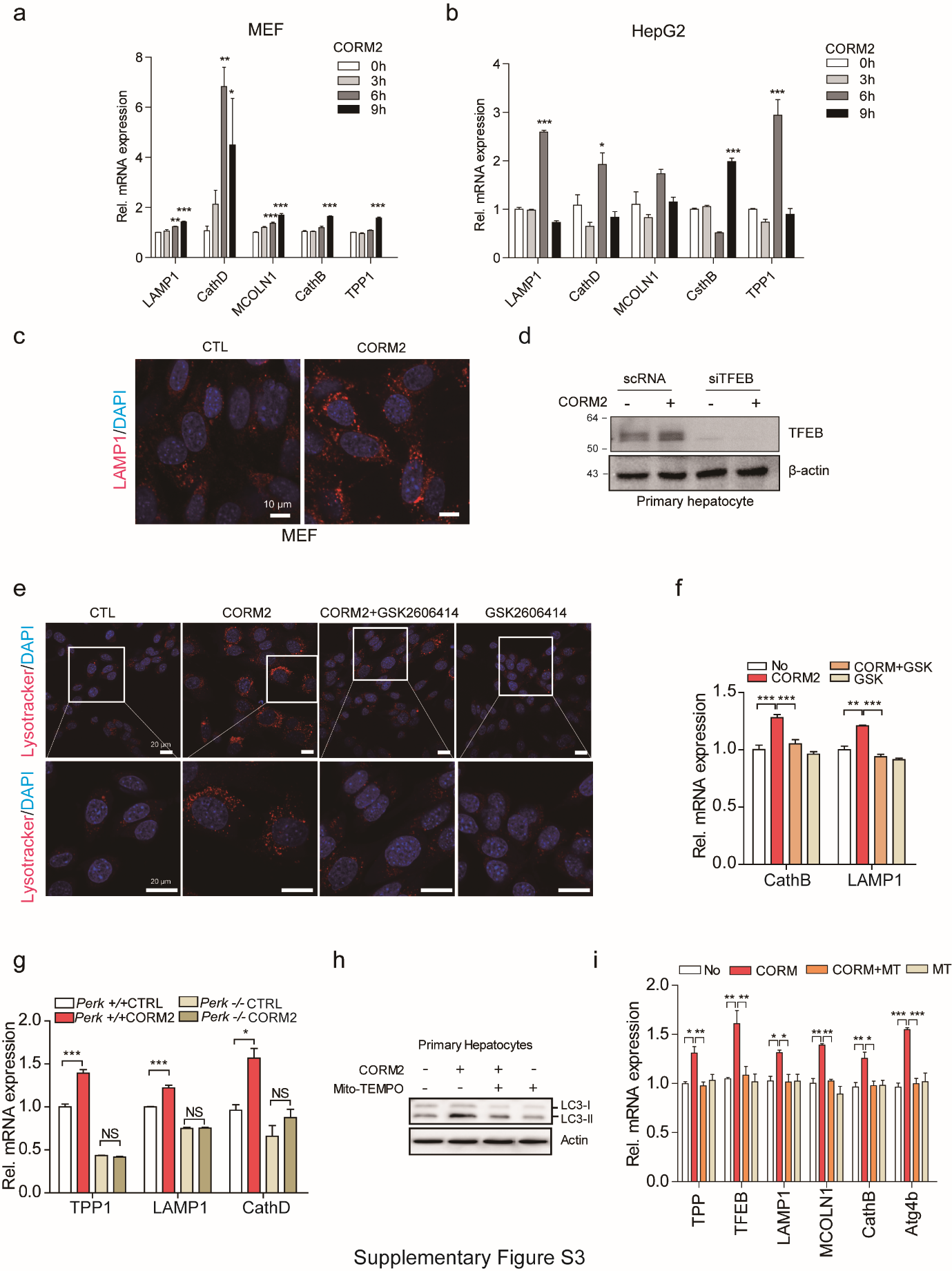
**

**Supplementary Fig. S3. CO promotes lysosomal biogenesis. (a, b)** MEFs and HepG2 cells were treated with CORM2 (20 μM) for indicated times. Total RNA was isolated and the expression of lysosomal genes, lysosomal-associated membrane protein 1 (LAMP-1), cathepsin D (CathD), mucolipin 1 (MCOLN1), cathepsin B (CathB), tripeptidyl-peptidase 1 (TPP1) were analyzed by qRT-PCR. Data are shown as mean ± SEM. **P*< 0.05, ***P*< 0.01. ****P*< 0.001. **(c)** *Perk^+/+^* MEFs were treated with CORM2 (20 μM) and then stained by anti-LAMP1 antibodies. Nuclei were stained with DAPI (blue). Scale bar, 10 μm. **(d)** Primary hepatocytes were transfected with scRNA or siTFEB for 24 hours, and treated with CORM2 (20 μM) for 6 hours. Knockdown of TFEB was confirmed by immunoblotting using TFEB antibody. **(e)** *Perk^+/+^* MEFs were transfected with scRNA or siTFEB for 24 hours and treated with CORM2 (20 μM) in the presence or absence of GSK2606414 as a PERK inhibitor. Cells were stained with Lysotracker Red staining. Lysosome puncta (red) were captured using a confocal fluorescence microscopy. Nuclei were stained with DAPI (blue). Scale bar, 20 μm.

**(f)** Primary hepatocytes pretreated with GSK2606414 were incubated with CORM2 (20 μM) for 24 hours. CathB, LAMP1 were analyzed by qRT-PCR. Data are shown as mean ± SEM. ***P*< 0.01. ****P*< 0.001 **(g)** *Perk^+/+^* MEF cells or *Perk^-/-^* MEF cells were incubated with CORM2 for 24 hours. TPP1, LAMP1, and CathD were analyzed by qRT-PCR. Data are shown as mean ± SEM. *P< 0.05, ***P*< 0.01. ****P*< 0.001 **(h-i)** Primary hepatocytes were treated with CORM2 (20 μM) for 6 h in the absence or presence of Mito-TEMPO (100 μM). After treatment, cells were lysed and analyzed by immunoblotting using anti-LC3 antibody (h) and qRT-PCR (i). Data are shown as mean ± SEM. *P< 0.05, **P< 0.01, ***P< 0.001.

**
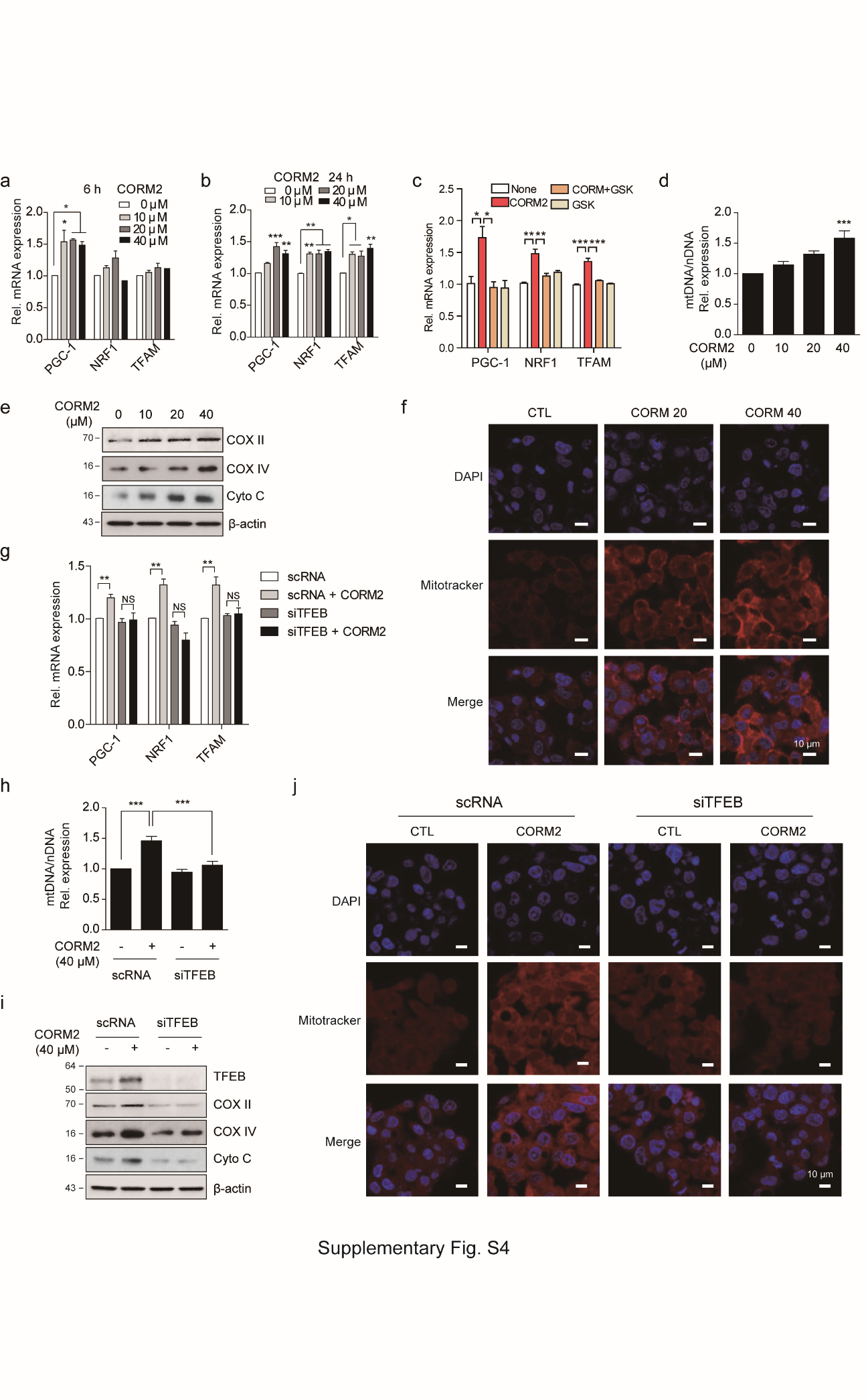
**

**Supplementary Fig. S4. CO induces mitochondrial biogenesis through TFEB-PGC1α pathway in HepG2 cells.** (**a-b, d-f**) HepG2 cells were treated with CORM2 at the indicated doses for 6 hours (**a**) or 24 hours (**b-f**). (**c**) Primary hepatocytes pretreated with GSK2606414 were incubated with CORM2 (20 μM) for 24 hours. (**g-j)** HepG2 cells were transfected with scRNA or siTFEB for 24 hours, and then treated with CORM2 for 24 hours. (**a, b, c g)** The expressions of PGC1α, NRF1 and TFAM were analyzed by qRT-PCR. Data are shown as mean ± SEM (n=3). *P< 0.05, **P < 0.01, ***P< 0.001. (**d, h**) The relative mtDNA content was measured by real-time PCR. mtDNA content was normalized to nDNA (β-actin gene) content. Data are shown as mean ± SEM (n=3). ***P< 0.001. (**e, i**) The levels of mitochondrial protein, complex II (COX II), complex IV (COX IV), and cytochrome *c* (Cyto *c*) were analyzed by immunoblotting. (**f**, **j**) Fluorescence intensity of MitoTracker Red (red) and DAPI (blue). Scale bar, 10 μm.
